# Evaluating prognostic block selection criteria in cervical medial branch radiofrequency neurotomy: A retrospective cohort study

**DOI:** 10.1016/j.inpm.2025.100559

**Published:** 2025-03-19

**Authors:** Allison Glinka Przybysz, Enrique Galang, Christian A. Sangio, Christian Wirawan, Amanda N. Cooper, Alycia Amatto, Brook Martin, Robert Burnham, Aaron M. Conger, Zachary L. McCormick, Taylor R. Burnham

**Affiliations:** a Department of Physical Medicine and Rehabilitation, University of Utah, Salt Lake City, UT, USA; b Department of Anesthesiology, Wake Forest University School of Medicine, Winston-Salem, NC, USA; c Wake Forest University School of Medicine, Winston-Salem, NC, USA; d Schwab Rehabilitation, University of Chicago, Chicago, IL, USA; e Department of Orthopedics, University of Utah Salt Lake City, UT, USA; f Division of Physical Medicine and Rehabilitation, Department of Medicine, University of Alberta, Edmonton, AB, Canada; g Central Alberta Pain and Rehabilitation Institute, Lacombe, AB, Canada; h Vivo Cura Health, Calgary, AB, Canada

**Keywords:** Radiofrequency ablation, Cervical facet pain, Selection criteria

## Abstract

**Background:**

Considerable variability exists in the literature record regarding patient selection criteria for cervical medial branch radiofrequency neurotomy (CMBRFN). Few prior studies have assessed the correlation between different prognostic block paradigms and treatment outcomes for this procedure.

**Objectives:**

Examine the association between various prognostic block selection criteria and CMBRFN success rates.

**Methods:**

Retrospective cohort study of consecutive patients from two Canadian musculoskeletal pain management clinics who underwent first-time CMBRFN between 2016 and 2022 with a three-tined cannula utilizing a perpendicular approach. Patients were categorized according to prognostic block paradigms (single vs. dual), block type (medial branch block [MBB] vs. intraarticular block [IAB]), and percentage pain relief after blocks. Six block criteria were established: 1 = MBB/MBB≥80 %; 2 = MBB/MBB 50–79 %; 3 = IAB/MBB≥80 %; 4 = IAB/MBB 50–79 %; 5 = MBB≥80 %; 6 = MBB 50–79 %. Treatment success was evaluated at 3 months post-CMBRFN as the proportion of participants with (1) ≥50 % NRS pain score reduction (the primary outcome) and (2) ≥17-point score decrease (the minimal clinically important difference [MCID]) on the Pain Disability Quality-of-Life Questionnaire – Spine (PDQQ-S). Logistic regression analyses were used to explore associations between block criteria and CMBRFN treatment success.

**Results:**

A total of 171 consecutive patients (58.5 % female; 58.0 ± 12.1 years of age; BMI 28.7 ± 6.0 kg/m^2^) were included. 60.8 % (95%CI: 53.3–67.8 %) and 61.4 % (95%CI: 53.9–68.7 %) of patients reported ≥50 % NRS and ≥17-point PDQQ-S reduction, respectively. After controlling for demographic factors, there were no statistically significant differences in the odds of treatment success amongst individuals selected by various prognostic block paradigms.

**Conclusion:**

Over 60 % of patients who underwent CMBRFN reported clinically significant magnitudes of improvement in pain and disability at three months post-CMBRFN, regardless of prognostic block selection criteria. These findings suggest that multiple block strategies might be employed to determine eligibility for CMBRFN. Larger, prospective studies including long-term outcome assessments are needed to further evaluate these findings.

## 1 Introduction

Neck pain disorders are a leading cause of disability in the United States [1]. Although the reported prevalence of facet joint-mediated neck pain varies widely, it is commonly estimated to impact up to 50 % of the general population [[2], [3], [4]]. As the incidence of chronic neck pain continues to escalate with aging populations, there is a growing need for targeted interventions to be included in the treatment paradigm. Cervical medial branch radiofrequency neurotomy (CMBRFN) is one such interventional procedure aimed at alleviating facet joint-derived pain by disrupting nociceptive signal conduction in the cervical medial branches of the dorsal rami, which innervate these neck joints [5].

Radiofrequency neurotomy, including CMBRFN, is regarded as an effective and safe therapeutic modality when patient selection is optimized [[6], [7], [8], [9]]. This procedure involves applying thermal energy to ablate the medial branch nerves at targeted levels, resulting in diminished pain transmission from the affected facet joints. However, despite its established efficacy, there is a notable paucity of literature regarding the specific prognostic indicators that optimize the prediction of positive outcomes for patients undergoing CMBRFN.

Currently, the most widely-accepted patient selection paradigms for this therapy are based on patient-reported symptomatic improvement following prognostic medial branch blocks [1,4]. However, the exact cutoffs of percentage improvement required for a positive block response vary considerably, with thresholds ranging from 50 % to 100 % reductions of index pain [10,11]. Previous studies that have categorized CMBRFN outcomes based on different prognostic block response thresholds show varying results; these studies report a range from no difference between thresholds to up to 58 % with a ≥80 % pain relief cutoff [10]. Determination of an optimal block paradigm and pain relief threshold may improve treatment responder rates and allow more patients to benefit from CMBRFN, particularly if lower thresholds prove as effective as higher ones [12,13].

This study addresses gaps in previous research by retrospectively analyzing CMBRFN outcomes in a cohort of patients selected for treatment with six distinct block criteria. It aims to identify the prognostic block selection criteria that most accurately predict successful pain relief and improvement in quality of life. These findings inform clinical decision-making related to optimizing patient selection for CMBRFN.

## 2 Methods

### 2.1 Data collection

Approval for this retrospective cohort study was granted by the Conjoint Health Research Ethics Board at the University of Calgary (Ethics ID#: REB20-0355). We identified consecutive patients from two Canadian musculoskeletal pain management clinics who underwent CMBRFN over a span of 6 years (2016–2022) through electronic medical record queries. Data extraction was performed by the authors (R.B. and A.A.) and included demographics and clinical variables such as age, gender, BMI, regular exercise, employment, smoking, and CMBRFN laterality.

Inclusion criteria were: (1) axial neck pain that was unresponsive to conventional conservative treatment; (2) clinical signs suggestive of cervical facet-mediated pain, such as pain and tenderness upon palpation over the cervical facet joints, or pain with cervical extension or rotation; (3) fluoroscopically guided prognostic blocks resulting in ≥50 % pain relief; (4) first-time CMBRFN procedure, excluding repeat procedures; (5) use of an 18-gauge Trident cannula with a 5-mm active tip (Diros Technology, Inc., Markham, Ontario, CA); and (6) Pain Disability and Quality of Life Questionnaire – Spine (PDQQ-S) assessments completed at baseline and 3 months post-intervention. For criterion (#3), the response to prognostic blocks was determined by having patients complete a 0–10 Numerical Rating Scale (NRS) pain diary. Recordings were made just prior to and at 30-min intervals for 6 h post-block, and this diary was sent back to our clinic for review. The maximal pain relief during this period was calculated from the pre-versus post-block NRS pain scores. Patients were included in the study if they achieved ≥50 % pain relief at any time within this 6-h period. Additionally, patients were asked if they experienced any functional improvement or enhanced ability to participate in specific activities, though this was not an inclusion criterion. The exclusion criteria were: (1) prognostic blocks performed off-site (2) confounding interventions or injuries (*e.g.*, epidural or facet joint steroid injection) occurring between the CMBRFN and follow-up; and (3) cervical procedures performed concurrently with another intervention on the same date.

### 2.2 Procedures

All procedures were performed by experienced interventional spine physicians. No intravenous sedation was used in any of the procedures.

#### 2.2.1 Prognostic blocks

##### 2.2.1.1 Medial branch blocks (MBBs)

Patients were positioned in the lateral recumbent or prone position (depending on provider preference). The skin was prepped and draped in sterile fashion. Using fluoroscopic guidance, 25-gauge, 1.5- to 2.5-inch short bevel needles were advanced to the appropriate anatomic landmarks to target the third occipital nerve (TON) and the C3–C7 medial branch nerves (MBNs). Correct needle placement was confirmed with anteroposterior (AP) and lateral views on fluoroscopy. Then, 0.3 mL of either 2 % lidocaine or 0.5 % bupivacaine was injected per site.

##### 2.2.1.2 Intraarticular facet joint injections (intraarticular blocks [IABs])

Patients were positioned in the lateral or prone positions (depending on provider preference and ease of facet joint accessibility). Cervical facet joints were injected using 22- or 25-gauge spinal needles (depending on body habitus). Needles were advanced under fluoroscopic guidance to enter the lateral or posterior facet joint space as confirmed by biplanar imaging. The injection was completed with 0.5 mL of 1 % preservative-free lidocaine and 2.5–5 mg of dexamethasone per joint.

##### 2.2.1.3 Radiofrequency ablation

**Fig. 1:**
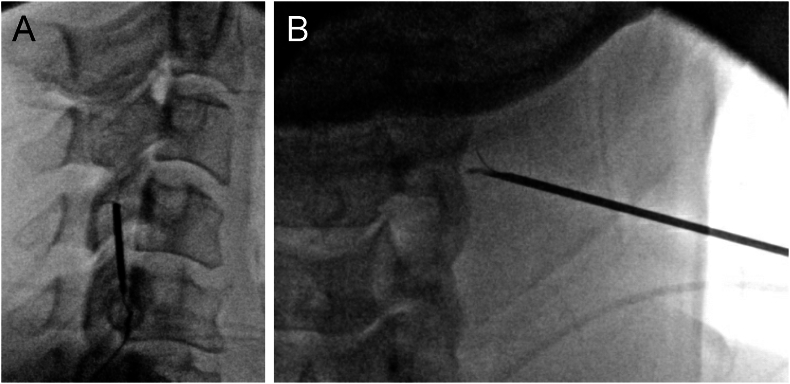
Lateral (A) and anteroposterior (B) fluoroscopic views of cervical medial branch radiofrequency neurotomy.

CMBRFN was performed using an 18-gauge multi-tined cannula with a 5-mm active tip (Diros Technology Inc., Markham, Ontario, Canada). Patients were positioned in the lateral decubitus position, and the cannula was inserted laterally into the neck to achieve perpendicular placement relative to the nerve. Cannula progression was monitored using lateral and AP fluoroscopic projections (Fig. 1). Once the target was reached, the tines of the cannula were deployed under direct visualization using the AP (to the patient) view. After optimal cannula placement was confirmed, 1–2 mL of 1–2% lidocaine was injected over each MBN. A single lesion was performed at each MBN, except for two lesions for the TON, by heating the electrode to 80 °C for 90s after a 15-s ramp up.

### 2.3 Outcome assessment

Patient selection paradigms were categorized based on the number (single vs. dual) and type (MBB vs. IAB) of prognostic blocks, as well as subsequent percentage pain relief based on patient-reported NRS pain scores collected pre- versus post-block. Percentage pain reduction from baseline in response to blocks was calculated using the lowest NRS pain score recorded within 6 h post-injection. Using this schema, six block criteria were established: (1) MBB/MBB ≥80 %, (2) MBB/MBB 50–79 %, (3) IAB/MBB ≥80 %, (4) IAB/MBB 50–79 %, (5) MBB ≥80 %, and (6) MBB 50–79 %. Outcomes relating to pain and function were respectively assessed using an 11-point NRS and the Pain Disability and Quality of Life Questionnaire – Spine (PDQQ-S), a six-item questionnaire validated for use as a patient-reported outcome measure within minimally invasive interventional spine care [14]. The minimal clinically important difference (MCID) determined for PDQQ-S is a score decrease of ≥17 points [15]. Treatment success was evaluated at 3 months post-CMBRFN using two categorical outcome measures: (1) a primary outcome of the proportion of participants with ≥50 % NRS pain score reduction and (2) a secondary outcome of the proportion of participants with ≥17-point decrease on PDQQ-S.

### 2.4 Statistical analyses

Data analysis included descriptive statistics, with means/standard deviations calculated for continuous variables and frequencies/percentages for categorical variables. Logistic regression models with calculated odds ratios (ORs) and their associated 95 % confidence intervals (CIs) were used to explore the relationships between the six block paradigms and treatment success while controlling for select demographic and procedural variables. Included covariates were gender, regular exercise, employment, smoking, CMBRFN laterality, age, and BMI.

## 3 Results

Of the 292 consecutive patients we identified, a total of 171 participants met eligibility criteria and were included in the analyses. Participant demographic, clinical, and procedural variables are presented in Table 1. This cohort was predominantly female (58.5 %) with a mean age of 58.0 ± 12.1 years and mean BMI of 28.7 ± 6.0 kg/m^2^. Distribution of the six block criteria was as follows: (1) 30.4 % MBB/MBB ≥80 %, (2) 32.2 % MBB/MBB 50–79 %, (3) 2.9 % IAB/MBB ≥80 %, (4) 5.3 % IAB/MBB 50–79 %, (5) 19.9 % MBB ≥80 %, and (6) 9.4 % MBB 50–79 %.

**Table 1:** Patient demographics, clinical characteristics, and procedure-related variables (*N* = 171).

Variable	No. (%)
Gender	
Male	71 (41.5)
Female	100 (58.5)
**Smoking**	
Yes	26 (18.4)
No	115 (81.6)
*Missing*	*30*
**Exercise**	
Yes	70 (50.0)
No	70 (50.0)
*Missing*	*31*
**Working**	
Yes	69 (50.4)
No	28 (20.4)
Retired	40 (29.2)
*Missing*	*34*
**Workup**	
Internal	143 (83.6)
External	28 (16.4)
**Clinic**	
Practice 1	66 (38.6)
Practice 2	105 (61.4)
**Prognostic block paradigm**	
MBB/MBB ≥80 %	52 (30.4)
MBB/MBB 50–79 %	55 (32.2)
IAB/MBB ≥80 %	5 (2.9)
IAB/MBB 50–79 %	9 (5.3)
MBB ≥80 %	34 (19.9)
MBB 50–79 %	16 (9.4)
**CMBRFN laterality**	
Unilateral	97 (56.7)
Bilateral	74 (43.3)

**Age in yr** (*n* = 171); mean (SD)	58.0 (12.1)
**Body mass index in kg/m**^**2**^ (*n* = 128); mean (SD)	28.7 (6.0)
**Pain duration in yr** (*n* = 135); mean (SD)	9.2 (10.5)

CMBRFN = cervical medial branch radiofrequency neurotomy; IAB = intraarticular block; MBB = medial branch block; SD = standard deviation.

At 3 months post-CMBRFN, the overall proportion of participants who reported ≥50 % NRS pain reduction was 60.8 % (95 % CI: 53.3–67.8 %), while 61.4 % (95 % CI: 53.9–68.7 %) reported a ≥17-point decrease (the MCID [15]) on PDQQ-S (Table 2). Logistic regression models for the primary and secondary study outcomes revealed no statistically significant associations between any of the six prognostic block paradigms and CMBRFN treatment success when controlling for select demographic and procedural covariates (Table 3).

**Table 2:** Primary and secondary study outcomes (*N* = 171).

Outcome	No. (%)	95 % CI
≥50 % NRS reduction		
Yes	104 (60.8)	53.3, 67.8
No	67 (39.2)	32.2, 46.7
≥17-point PDQQ-S reduction		
Yes	105 (61.4)	53.9, 68.7
No	66 (38.6)	31.6, 46.1

CI = confidence interval; NRS = Numerical Rating Scale; PDQQ-S = Pain Disability and Quality-of-Life Questionnaire – Spine.

**Table 3:** Logistic regression models for ≥50 % NRS reduction and ≥17-point PDQQ-S reduction.

Outcome	Predictor	OR	95 % CI	*p*
≥50 % NRS reduction^a^	**Gender** (vs. male)			
	Female	1.08	0.45, 2.61	0.86
	**Exercise** (vs. no)			
	Yes	0.98	0.42, 2.30	0.97
	**Working** (vs. not working)			
	Yes	0.76	0.27, 2.17	0.61
	Retired	1.12	0.32, 3.96	0.86
	**Smoking** (vs. no)			
	Yes	0.71	0.26, 1.96	0.51
	**Laterality** (vs. unilateral)			
	Bilateral	1.40	0.64, 3.05	0.39
	**Block type** (vs. MBB/MBB ≥80 %)			
	MBB/MBB 50–79 %	0.76	0.27, 2.12	0.60
	IAB/MBB ≥80 %	1.62	0.10, 25.91	0.73
	IAB/MBB 50–79 %	0.53	0.10, 2.70	0.44
	MBB ≥80 %	0.73	0.22, 2.35	0.59
	MBB 50–79 %	0.39	0.09, 1.62	0.20
	**Age**	0.97	0.93, 1.01	0.20
	**BMI**	1.05	0.98, 1.13	0.20

≥17-point PDQQ-S reduction^b^	**Gender** (vs. male)			
	Female	1.51	0.65, 3.52	0.34
	**Exercise** (vs. no)			
	Yes	1.08	0.48, 2.46	0.85
	**Working** (vs. not working)			
	Yes	0.64	0.22, 1.86	0.41
	Retired	0.85	0.23, 3.14	0.80
	**Smoking** (vs. no)			
	Yes	0.61	0.22, 1.66	0.33
	**Laterality** (vs. unilateral)			
	Bilateral	1.19	0.54, 2.59	0.67
	**Block type** (vs. MBB/MBB ≥80 %)			
	MBB/MBB 50–79 %	0.72	0.27, 1.91	0.51
	IAB/MBB ≥80 %	1.48	0.13, 16.27	0.75
	IAB/MBB 50–79 %	0.54	0.12, 2.48	0.43
	MBB ≥80 %	1.41	0.40, 4.91	0.59
	MBB 50–79 %	0.70	0.16, 3.07	0.63
	**Age**	0.99	0.95, 1.03	0.49
	**BMI**	0.99	0.93, 1.07	0.89

CI = confidence interval; IAB = intraarticular block; MBB = medial branch block; NRS = Numerical Rating Scale; OR = odds ratio; PDQQ-S = Pain Disability and Quality-of-Life Questionnaire – Spine.

a *N* = 121; *χ*^2^ (13) = 8.31; *p* = 0.82; Pseudo *R*^2^ = 0.05.

b *N* = 121; *χ*^2^ (13) = 6.04; *p* = 0.94; Pseudo *R*^2^ = 0.04.

To mitigate concerns for adequate statistical power given that the sample distribution was comparatively small for three of the six block criteria (*n* = 5 IAB/MBB ≥80 %; *n* = 9 IAB/MBB 50–79 %; *n* = 16 MBB 50–79 %), we performed supplemental logistic regression analyses for treatment success with block criteria reorganized in four different ways. Specifically, categories were regrouped by (1) combining IAB- and MBB-based block criteria and (2) removing IAB-based criteria, as well as examining (3) single versus dual blocks and (4) blocks with 50–79 % versus ≥80 % pain relief. Results of these analyses are presented in Appendices A–D. Briefly, there was no change in the final results associated with block paradigms.

## 4 Discussion

The primary objective of the present study was to determine the associations between various commonly utilized block-based selection paradigms and treatment success with CMBRFN using a three-tined (Trident) cannula positioned perpendicular to the medial branch nerve. Our findings demonstrate that most of those who underwent CMBRFN achieved clinically significant pain relief and functional improvement at 3 months. Approximately 61 % of participants reported ≥50 % reductions in NRS scores and ≥17-point reductions in PDQQ-S scores, regardless of the prognostic block selection criteria. According to logistic regression models, none of the six block criteria or other covariates we examined significantly influenced the likelihood of treatment success following CMBRFN.

Our findings align with previous descriptions of 3-month CMBRFN success rates, which range from 63 to 75 % for patients selected via dual MBBs (± a third placebo block with saline) requiring 100 % symptom relief [[16], [17], [18]]. A 1999 study by McDonald et al. found that 18 of 28 (64 %) patients experienced ≥50 % pain reduction lasting at least 3 months post-procedure [16]. The majority of patient-reported outcome data were collected previously by the investigators as part of two earlier prospective trials. In 1995, Lord et al. published 3-month outcomes of a feasibility study in which 12 of 19 patients (63 %) had complete pain relief [17]. Similar results were obtained in the ensuing randomized clinical trial, where 9 out of 12 (75 %) participants were symptom-free at 3 months post-CMBRFN [18].

Interestingly, favorable patient outcomes from these investigations by Lord and colleagues helped to inform the current International Pain and Spine Intervention Society (IPSIS) Guidelines recommendation of complete symptom relief with dual MBBs ± a third placebo block as the standard patient selection criterion for CMBRFN [5]. According to a recent pooled analysis by Engel et al., 52 % (95 % CI: 40–64 %) of patients selected with this criterion went on to experience complete relief of symptoms at 6 months post-CMBRFN [19]. While this approach reduces false-positive rates [20], it is not a current requirement for insurance coverage by most payer types in the USA [21]. Furthermore, such a strict selection protocol may create unnecessary barriers to treatment by restricting access from potential responders while imposing greater patient costs and time requirements to qualify for a relatively safe procedure (CMBRFN).

In community-based practice, patients are commonly selected for CMBRFN using the current IPSIS standard for lumbar MBRFN (dual concordant MBBs requiring ≥80 % symptom improvement) [22], but this has largely been driven by Medicare requirements. Expert contributors to more recently published practice guidelines have also questioned the need for stringent CMBRFN selection paradigms. A set of multi-society, consensus-based guidelines published in 2022 by Hurley et al. recommend an even less restrictive paradigm of ≥50 % relief with a single MBB [10].

Few published clinical research reports to date have investigated the correlations between various prognostic block criteria and CMBRFN treatment success. Intraarticular facet joint blocks (IABs) have not been validated, making MBBs the current gold standard for diagnosing facet-mediated neck pain [5]. However, limited available evidence suggests that CMBRFN treatment outcomes do not appear to suffer when IAB-based criteria are used to select patients. When sharing results of a cross-sectional cohort study, Speldewinde et al. noted that 112 of 151 (76 %) patients selected for CMBRFN via dual MBBs and/or IABs requiring ≥80 % relief met the responder definition of ≥50 % pain reduction for at least at 2 months post-procedure [23]. Smith et al. found that pain was significantly reduced from baseline by 45 % at 3 months post-CMBRFN in patients selected by dual concordant IAB/MBB injections requiring ≥50 % relief [24].

MBB-based prognostic block paradigms may be performed on a single or double basis, with dual concordant MBBs requiring higher cutoff thresholds for pain relief thought to decrease false positives and improve CMBRFN success rates [19,25]. However, the extent to which additional MBBs affect CMBRFN outcomes in real-world practice is currently unclear. Cadwell et al. shared a recent retrospective analysis comparing CMBRFN treatment outcomes between patients selected by either single (*n* = 41) or dual MBBs (*n* = 28) with a post-block threshold of ≥80 % pain relief [26]. The authors found no significant between-group differences in 3-month responder rates for pain reduction, regardless of whether the relief threshold was set at ≥50 % (54 % single vs. 35 % dual) or ≥80 % (32 % single vs. 28 % dual). Likewise, evidence suggests that MBB paradigms with higher pain relief cutoff values may not significantly influence treatment success. A multi-center analysis of factors associated with CMBRFN outcomes by Cohen et al. found that success rates (defined as ≥50 % pain reduction for at least 6 months) did not differ between patients selected with single MBBs using block cutoffs of ≥50–79 % (30/54 patients; 56 %) or ≥80 % symptom relief (21/36 patients; 58 %) [27]. In a retrospective cross-sectional cohort study of patients selected with dual concordant MBBs, Burnham et al. found no difference in responder rates for ≥50 % pain reduction at a minimum of 6 (mean 16.9 ± 12.7) months post-CMBRFN when the block relief threshold was ≥80–99 % (14/26 patients; 54 % [95 % CI: 35–73 %]) or complete pain relief (13/24 patients; 54 % [95 % CI: 32–74 %]) [4].

Accumulating evidence suggests that more stringent block-based patient selection paradigms may not influence CMBRFN outcomes, which has implications for clinical practice. Our results showed that over 60 % of patients whose index pain is reduced by ≥ 50 % with at least one MBB went on to experience clinically significant improvements to pain and function at 3 months post-CMBRFN, regardless of whether a positive response to an initial intraarticular injection with steroid/anesthetic or a second MBB was required. Additionally, logistic regression models revealed that none of the six block criteria were significantly associated with treatment success while controlling for select demographic and procedural covariates. These observations and previous studies challenge the putative need for stringent block-based selection criteria. Further research is warranted to evaluate the prognostic value of selection criteria and other demographic and clinical factors for predicting CMBRFN success. Future investigations should ideally include RCTs with long-term follow-up featuring large patient populations.

### 4.1 Strengths and limitations

The strengths of this study lie in its novelty, large sample size, and use of real-world data. To our knowledge, the present evaluation of CMBRFN patient selection paradigms is more comprehensive than any literature reports for a single practice to date. We examined six distinct block criteria distinguished according to block type, number, and degree of pain relief in a sizeable cohort of 171 patients. This level of diversity in CMBRFN selection protocols is unique among published records of large-scale investigations, including individual practice audits [23] and single- and multi-center analyses [4,27]. Lastly, utilizing real-world data from community-based practice improves the external validity of this research, providing a more accurate and relevant context compared to controlled experimental settings.

Our study is not without limitations. With a single-arm retrospective cohort design, the ability to definitively assess the outcomes of CMBRFN with its respective block paradigm is limited as there is no control group. In addition, the study's retrospective nature precludes the adjustment of potential confounding variables. Moreover, our study presents short-term outcomes collected only at 3 months post-procedure, posing that selection-based disparities in CMBRFN outcomes could emerge with a longer follow-up time frame. Finally, the cannula (three-tined Trident probe) and approach to the medial branch nerve (perpendicular) used in the present study are still relatively new and not routinely utilized in clinical practice or the research cited in this paper. This may limit the generalizability of our findings. However, the popularity of this technique is increasing as early research exploring its effectiveness yields promising results, although one study found pain relief duration to be longer when the conventional cannula/parallel to the medial branch nerve technique was used [[28], [29], [30], [31]].

## 5 Conclusion

Over 60 % of patients who underwent first-time CMBRFN reported clinically significant improvements in pain and disability at 3 months post-procedure, regardless of prognostic block selection criteria, as long as they experienced ≥50 % pain reduction with at least one prognostic MBB. These findings suggest that multiple block strategies might be employed to determine eligibility for CMBRFN. Larger prospective studies with an assessment of long-term outcomes are needed to further evaluate these findings.

## Funding

None.

## Declaration of competing interest

The authors declare the following financial interests/personal relationships which may be considered as potential competing interests: Zachary McCormick reports a relationship with International Pain and Spine Intervention Society that includes: board membership. Zachary McCormick reports a relationship with Avanos Medical Inc that includes: consulting or advisory and funding grants. Zachary McCormick reports a relationship with Boston Scientific Corporation that includes: funding grants. Zachary McCormick reports a relationship with Relievant Medsystems Inc that includes: funding grants. Zachary McCormick reports a relationship with Saol Therapeutics that includes: consulting or advisory and funding grants. Zachary McCormick reports a relationship with Spine Biopharma that includes: funding grants. Zachary McCormick reports a relationship with SPR Therapeutics Inc that includes: funding grants. Zachary McCormick reports a relationship with Stratus Medical that includes: funding grants. Zachary McCormick reports a relationship with Stryker that includes: consulting or advisory. Zachary McCormick reports a relationship with OrthoSon that includes: consulting or advisory. Taylor Burnham reports a relationship with Diros Technology Inc that includes: funding grants. Aaron Conger reports a relationship with Stratus that includes: funding grants. If there are other authors, they declare that they have no known competing financial interests or personal relationships that could have appeared to influence the work reported in this paper.

## Figures and Tables

**Table A1:** Alternate categorization of block paradigms by combining IAB- and MBB-based criteria

Prognostic block paradigm	No. (%)
IABs combined with MBBs (*n* = 171)	
Dual ≥80 %	57 (33.3)
Dual 50–79 %	64 (37.4)
Single ≥80 %	34 (19.9)
Single 50–79 %	16 (9.4)

IAB = intraarticular block; MBB = medial branch block.

**Table A2:** Univariate logistic regression models for treatment outcomes with IAB- and MBB-based criteria combined

Outcome	Predictor	OR	95 % CI	*P*
≥50 % NRS reduction^a^	**Block type** (vs. dual ≥80 %)			
	Dual 50–79 %	0.73	0.35, 1.54	0.41
	Single ≥80 %	0.81	0.33, 1.96	0.64
	Single 50–79 %	0.39	0.13, 1.21	0.10

≥17-point PDQQ-S reduction^b^	**Block type** (vs. dual ≥80 %)			
	Dual 50–79 %	0.64	0.31, 1.35	0.24
	Single ≥80 %	1.05	0.42, 2.59	0.92
	Single 50–79 %	0.50	0.16, 1.54	0.23

CI = confidence interval; IAB = intraarticular block; MBB = medial branch block; NRS = Numerical Rating Scale; OR = odds ratio; PDQQ-S = Pain Disability and Quality-of-Life Questionnaire – Spine.

a *N* = 171; *χ*^2^ (3) = 2.75; *p* = 0.43; Pseudo *R*^2^ = 0.01.

b *N* = 171; *χ*^2^ (3) = 2.78; *p* = 0.43; Pseudo *R*^2^ = 0.01.

**Table A3:** Multiple logistic regression models for treatment outcomes with IAB- and MBB-based criteria combined

Outcome	Predictor	OR	95 % CI	*P*
≥50 % NRS reduction^a^	**Block number** (vs. dual ≥80 %)			
	Dual 50–79 %	0.68	0.26, 1.80	0.44
	Single ≥80 %	0.70	0.22, 2.21	0.54
	Single 50–79 %	0.37	0.09, 1.54	0.17
	**Gender** (vs. male)			
	Female	1.06	0.44, 2.54	0.89
	**Smoking** (vs. no)			
	Yes	0.70	0.26, 1.92	0.49
	**Exercise** (vs. no)			
	Yes	0.96	0.41, 2.25	0.93
	**Working** (vs. not working)			
	Yes	0.73	0.26, 2.03	0.55
	Retired	1.09	0.31, 3.80	0.89
	**Laterality** (vs. unilateral)			
	Bilateral	1.41	0.64, 3.08	0.39
	**Age**	0.97	0.93, 1.01	0.17
	**BMI**	1.05	0.97, 1.13	0.21

≥17-point PDQQ-S reduction^b^	**Block number** (vs. dual ≥80 %)			
	Dual 50–79 %	0.66	0.27, 1.65	0.37
	Single ≥80 %	1.37	0.40, 4.68	0.62
	Single 50–79 %	0.67	0.16, 2.88	0.59
	**Gender** (vs. male)			
	Female	1.48	0.64, 3.44	0.36
	**Smoking** (vs. no)			
	Yes	0.60	0.22, 1.65	0.33
	**Exercise** (vs. no)			
	Yes	1.06	0.47, 2.41	0.88
	**Working** (vs. not working)			
	Yes	0.62	0.22, 1.78	0.38
	Retired	0.83	0.23, 3.04	0.78
	**Laterality** (vs. unilateral)			
	Bilateral	1.19	0.54, 2.62	0.66
	**Age**	0.98	0.95, 1.03	0.46
	**BMI**	0.99	0.93, 1.07	0.86

CI = confidence interval; IAB = intraarticular block; MBB = medial branch block; NRS = Numerical Rating Scale; OR = odds ratio; PDQQ-S = Pain Disability and Quality-of-Life Questionnaire – Spine.

a *N* = 121; *χ*^2^ (11) = 8.03; *p* = 0.71; Pseudo *R*^2^ = 0.05.

b *N* = 121; *χ*^2^ (11) = 5.67; *p* = 0.89; Pseudo *R*^2^ = 0.03.

**Table B1:** Alternate categorization of block paradigms with IAB-based criteria removed

Prognostic block paradigm	No. (%)
IABs removed (*n* = 157)	
MBB/MBB ≥80 %	52 (33.1)
MBB/MBB 50–79 %	55 (35.0)
MBB ≥80 %	34 (21.7)
MBB 50–79 %	16 (10.2)

IAB = intraarticular block; MBB = medial branch block.

**Table B2:** Univariate logistic regression models for treatment outcomes with IAB-based criteria removed

Outcome	Predictor	OR	95 % CI	*P*
≥50 % NRS reduction^a^	**Block type** (vs. MBB/MBB ≥80 %)			
	MBB/MBB 50–79 %	0.86	0.39, 1.89	0.70
	MBB ≥80 %	0.86	0.35, 2.10	0.73
	MBB 50–79 %	0.41	0.13, 1.29	0.13

≥17-point PDQQ-S reduction^b^	**Block type** (vs. MBB/MBB ≥80 %)			
	MBB/MBB 50–79 %	0.74	0.34, 1.62	0.45
	MBB ≥80 %	1.11	0.44, 2.78	0.83
	MBB 50–79 %	0.53	0.17, 1.65	0.27

CI = confidence interval; IAB = intraarticular block; MBB = medial branch block; NRS = Numerical Rating Scale; OR = odds ratio; PDQQ-S = Pain Disability and Quality-of-Life Questionnaire – Spine.

a *N* = 157; *χ*^2^ (3) = 2.35; *p* = 0.50; Pseudo *R*^2^ = 0.01.

b *N* = 157; *χ*^2^ (3) = 1.99; *p* = 0.57; Pseudo *R*^2^ = 0.01.

**Table B3:** Multiple logistic regression models for treatment outcomes with IAB-based criteria removed

Outcome	Predictor	OR	95 % CI	*P*
≥50 % NRS reduction^a^	**Block type** (vs. MBB/MBB ≥80 %)			
	MBB/MBB 50–79 %	0.79	0.28, 2.25	0.66
	MBB ≥80 %	0.77	0.23, 2.52	0.66
	MBB 50–79 %	0.39	0.09, 1.65	0.20
	**Gender** (vs. male)			
	Female	1.09	0.43, 2.77	0.85
	**Smoking** (vs. no)			
	Yes	0.57	0.20, 1.62	0.29
	**Exercise** (vs. no)			
	Yes	0.84	0.34, 2.09	0.71
	**Working** (vs. not working)			
	Yes	0.82	0.27, 2.45	0.72
	Retired	1.06	0.27, 4.24	0.93
	**Laterality** (vs. unilateral)			
	Bilateral	1.44	0.63, 3.29	0.39
	**Age**	0.97	0.93, 1.01	0.18
	**BMI**	1.05	0.97, 1.13	0.26

≥17-point PDQQ-S reduction^b^	**Block type** (vs. MBB/MBB ≥80 %)			
	MBB/MBB 50–79 %	0.71	0.27, 1.91	0.50
	MBB ≥80 %	1.43	0.42, 4.82	0.57
	MBB 50–79 %	0.64	0.15, 2.75	0.55
	**Gender** (vs. male)			
	Female	1.25	0.51, 3.07	0.62
	**Smoking** (vs. no)			
	Yes	0.67	0.24, 1.92	0.46
	**Exercise** (vs. no)			
	Yes	0.99	0.42, 2.37	0.99
	**Working** (vs. not working)			
	Yes	0.91	0.30, 2.77	0.87
	Retired	1.03	0.26, 4.169	0.97
	**Laterality** (vs. unilateral)			
	Bilateral	1.12	0.50, 2.53	0.78
	**Age**	0.99	0.94, 1.03	0.51
	**BMI**	1.00	0.93, 1.08	1.00

CI = confidence interval; IAB = intraarticular block; MBB = medial branch block; NRS = Numerical Rating Scale; OR = odds ratio; PDQQ-S = Pain Disability and Quality-of-Life Questionnaire – Spine.

a *N* = 109; *χ*^2^ (11) = 8.06; *p* = 0.71; Pseudo *R*^2^ = 0.06.

b *N* = 109; *χ*^2^ (11) = 3.72; *p* = 0.98; Pseudo *R*^2^ = 0.02.

**Table C1:** Alternate categorization of block paradigms by number of blocks (single vs. dual)

Prognostic block paradigm	No. (%)
Number of blocks (*n* = 171)	
Dual	121 (70.8)
Single	50 (29.4)

**Table C2:** Univariate logistic regression models for treatment outcomes based on number of blocks (single vs. dual)

Outcome	Predictor	OR	95 % CI	*P*
≥50 % NRS reduction^a^	**Number of blocks** (vs. dual)			
	Single	0.75	0.39, 1.47	0.41
≥17-point PDQQ-S reduction^b^	**Number of blocks** (vs. dual)			
	Single	1.04	0.52, 2.05	0.92

CI = confidence interval; NRS = Numerical Rating Scale; OR = odds ratio; PDQQ-S = Pain Disability and Quality-of-Life Questionnaire – Spine.

a *N* = 171; *χ*^2^ (1) = 0.68; *p* = 0.41; Pseudo *R*^2^ = 0.00.

b *N* = 171; *χ*^2^ (1) = 0.01; *p* = 0.92; Pseudo *R*^2^ = 0.00.

**Table C3:** Multiple logistic regression models for treatment outcomes based on number of blocks (single vs. dual)

Outcome	Predictor	OR	95 % CI	*P*
≥50 % NRS reduction^a^	**Number of blocks** (vs. dual)			
	Single	0.69	0.29, 1.64	0.40
	**Gender** (vs. male)			
	Female	1.19	0.52, 2.71	0.67
	**Smoking** (vs. no)			
	Yes	0.69	0.25, 1.87	0.46
	**Exercise** (vs. no)			
	Yes	0.99	0.43, 2.28	0.98
	**Working** (vs. not working)			
	Yes	0.70	0.25, 1.94	0.49
	Retired	1.02	0.30, 3.43	0.97
	**Laterality** (vs. unilateral)			
	Bilateral	1.38	0.64, 2.97	0.41
	**Age**	0.97	0.93, 1.01	0.18
	**BMI**	1.04	0.97, 1.12	0.25

≥17-point PDQQ-S reduction^b^	**Number of blocks** (vs. dual)			
	Single	1.31	0.52, 3.33	0.57
	**Gender** (vs. male)			
	Female	1.67	0.74, 3.75	0.22
	**Smoking** (vs. no)			
	Yes	0.59	0.22, 1.59	0.30
	**Exercise** (vs. no)			
	Yes	1.09	0.49, 2.45	0.83
	**Working** (vs. not working)			
	Yes	0.61	0.22, 1.69	0.34
	Retired	0.78	0.23, 2.71	0.70
	**Laterality** (vs. unilateral)			
	Bilateral	1.16	0.53, 2.50	0.71
	**Age**	0.99	0.95, 1.03	0.47
	**BMI**	0.99	0.92, 1.06	0.79

CI = confidence interval; NRS = Numerical Rating Scale; OR = odds ratio; PDQQ-S = Pain Disability and Quality-of-Life Questionnaire – Spine.

a *N* = 121; *χ*^2^ (9) = 6.47; *p* = 0.69; Pseudo *R*^2^ = 0.04.

b *N* = 121; *χ*^2^ (9) = 3.81; *p* = 0.92; Pseudo *R*^2^ = 0.02.

**Table 1D:** Alternate categorization of block paradigms by percentage pain relief (50–79 % vs. ≥80 %)

Prognostic block paradigm	No. (%)
Block pain relief (*n* = 171)	
50–79 %	80 (46.8)
≥80 %	91 (53.2)

**Table 2D:** Univariate logistic regression models for treatment outcomes based on block percentage pain relief (50–79 % vs. ≥80 %)

Outcome	Predictor	OR	95 % CI	*P*
≥50 % NRS reduction^a^	**Block pain relief** (vs. 50–79 %)			
	≥80 %	1.43	0.77, 2.66	0.25
≥17-point PDQQ-S reduction^b^	**Block pain relief** (vs. 50–79 %)			
	≥80 %	1.66	0.89, 3.10	0.11

CI = confidence interval; NRS = Numerical Rating Scale; OR = odds ratio; PDQQ-S = Pain Disability and Quality-of-Life Questionnaire – Spine.

a *N* = 171; *χ*^2^ (1) = 1.31; *p* = 0.25; Pseudo *R*^2^ = 0.01.

b *N* = 171; *χ*^2^ (1) = 2.57; *p* = 0.11; Pseudo *R*^2^ = 0.01.

**Table 3D:** Multiple logistic regression models for treatment outcomes based on block percentage pain relief (50–79 % vs. ≥80 %)

Outcome	Predictor	OR	95 % CI	*P*
≥50 % NRS reduction^a^	**Block pain relief** (vs. 50–79 %)			
	≥80 %	1.45	0.66, 3.19	0.36
	**Gender** (vs. male)			
	Female	1.11	0.47, 2.61	0.81
	**Smoking** (vs. no)			
	Yes	0.68	0.25, 1.87	0.46
	**Exercise** (vs. no)			
	Yes	1.03	0.45, 2.33	0.95
	**Working** (vs. not working)			
	Yes	0.72	0.26, 2.01	0.53
	Retired	1.05	0.31, 3.58	0.94
	**Laterality** (vs. unilateral)			
	Bilateral	1.41	0.65, 3.07	0.38
	**Age**	0.97	0.93, 1.01	0.16
	**BMI**	1.04	0.97, 1.11	0.27

≥17-point PDQQ-S reduction^b^	**Block pain relief** (vs. 50–79 %)			
	≥80 %	1.69	0.78, 3.66	0.18
	**Gender** (vs. male)			
	Female	1.51	0.66, 3.45	0.33
	**Smoking** (vs. no)			
	Yes	0.63	0.23, 1.70	0.36
	**Exercise** (vs. no)			
	Yes	1.06	0.48, 2.36	0.88
	**Working** (vs. not working)			
	Yes	0.62	0.22, 1.75	0.36
	Retired	0.86	0.25, 3.01	0.82
	**Laterality** (vs. unilateral)			
	Bilateral	1.18	0.54, 2.57	0.68
	**Age**	0.98	0.95, 1.02	0.47
	**BMI**	1.00	0.93, 1.07	0.95

CI = confidence interval; NRS = Numerical Rating Scale; OR = odds ratio; PDQQ-S = Pain Disability and Quality-of-Life Questionnaire – Spine.

a *N* = 121; *χ*^2^ (9) = 7.48; *p* = 0.59; Pseudo *R*^2^ = 0.04.

b *N* = 121; *χ*^2^ (9) = 5.53; *p* = 0.79; Pseudo *R*^2^ = 0.03.
